# Experimental Setup for the Validation of Photoplethysmography Devices for the Evaluation of Arteriovenous Fistulas

**DOI:** 10.3390/bioengineering12090990

**Published:** 2025-09-18

**Authors:** Simone Chiorboli, Adriano Brugnoli, Vincenzo Piemonte

**Affiliations:** Unit of Chemical-Physics Fundamentals in Chemical Engineering, Department of Engineering, University Campus Bio-Medico of Rome, Via Álvaro del Portillo, 21, 00128 Roma, Italy; adriano.brugnoli00@gmail.com (A.B.); v.piemonte@unicampus.it (V.P.)

**Keywords:** hemodialysis, vascular access, arteriovenous fistula, stenosis, photoplethysmography, phantoms

## Abstract

This study describes the design and validation of an experimental setup for testing photoplethysmographic (PPG) devices intended for the non-invasive monitoring of vascular accesses in hemodialysis patients. Continuous assessment of arteriovenous fistulas is essential to detect pathological conditions such as stenosis, which can compromise patient safety and dialysis efficacy. While PPG-based sensors are capable of detecting such anomalies, their clinical applicability must be supported by controlled in vitro validation. The developed system replicates the anatomical, mechanical, optical, and hemodynamic features of vascular accesses. A 3D fistula model was designed and fabricated via 3D printing and silicone casting. The hydraulic circuit used red India ink and a PWM-controlled pump to simulate physiological blood flow, including stenotic conditions. Quantitative validation confirmed anatomical accuracy within 0.1 mm tolerance. The phantom exhibited an average Shore A hardness of 20.3 ± 1.1, a Young’s modulus of 10.4 ± 0.9 MPa, and a compression modulus of 105 MPa—values consistent with soft tissue behavior. Burst pressure exceeded 2000 mmHg, meeting ISO 7198:2016 standards. Flow rates (400–700 mL/min) showed <1% error. Compliance was 2.4 ± 0.2, and simulated blood viscosity was 3.9 ± 0.3 mPa·s. Systolic and diastolic pressures fell within physiological ranges. Photoplethysmographic signals acquired using a MAX30102 sensor (Analog devices Inc., Wilmington, MA, USA) reproduced key components of in vivo waveforms, confirming the system’s suitability for device testing.

## 1. Introduction

Vascular access represents the connection point between the patient’s circulatory system and the dialysis device circuit. It enables the withdrawal and return of blood during dialysis, facilitating effective extracorporeal circulation. Due to this access, blood can be properly purified from toxins through the dialysis process. There are different types of vascular accesses in hemodialysis, outlined as follows: arteriovenous fistula (AVF), arteriovenous graft, and central venous catheter.

All three types of vascular access play a fundamental role in the effectiveness of hemodialysis treatment, as they guarantee a high blood flow, which is essential for optimal blood purification during the dialysis process. An arteriovenous fistula is the connection of an artery and a vein. The most common site where the fistula is created is between the brachial artery and the cephalic vein. The arteriovenous fistula is created through surgery, followed by a maturation phase, during which the vein adapts to the higher pressure coming from the artery. After this adaptation phase, which allows the vein to become sufficiently resistant and dilated, the fistula is ready to be used for dialysis sessions. For dialysis treatment, two needles are inserted at the level of the arteriovenous fistula that are distant from each other as follows: one for the collection of blood to be treated with the machine and the other for the reintroduction of the detoxified blood into the bloodstream. This type of access is the most used and preferred for dialysis compared to the previous ones because it guarantees a longer duration and is less prone to infections. Although AVFs are less prone to infection, stenosis remains their most common complication. Stenosis is the narrowing of a vessel. In the context of hemodialysis, stenosis is the narrowing of the diameter of the arteriovenous fistula caused by the accumulation of calcium salts, cells, and scar tissue. More precisely, stenosis occurs when the narrowing of the lumen of the vein is greater than 50% of the diameter of the vessel [1,2,3,4,5,6,7,8,9]. The onset of stenosis is mainly caused by surgical stress due to the repeated venipunctures necessary to insert the needles during dialysis treatment; these frequent traumas and inflammations, at the level of the arteriovenous fistula, create endothelial lesions and the consequent narrowing of the vessel [10]. Stenosis has a progressive evolution that may lead a gradual worsening over time due to which, in very critical conditions, a stoppage of the blood flow can occur. This condition, therefore, is very dangerous to the patient, as it could also favor the formation of thrombi or emboli. The KDOQI foundation considers that a flow rate less than 600 mL/min is a symptom of stenotic vascular degeneration. For these reasons, continuous monitoring is essential to promptly detect any complications.

The AVF surveillance program aims to detect dysfunction early through regular monitoring, thereby preventing complications related to stenosis. Diagnostic tools used to diagnose arteriovenous fistula pathologies include doppler ultrasound [11], angiography, and photoplethysmography (PPG).

Photoplethysmography is a non-invasive, low-cost optical diagnostic technique that is widely used to monitor cardiovascular parameters such as the heart rate and oxygen saturation [12]. The operating principle of PPG is based on the interaction of light (usually red or infrared) with biological tissue [13]. A LED emits a beam of light that penetrates the tissue and is partially absorbed and reflected by the blood. A photodiode detects the amount of light transmitted or reflected, and the variation in its intensity is converted into an electrical signal [14,15]. PPG signal analysis allows us to identify hemodynamic alterations compatible with the presence of stenosis [16]. A healthy fistula produces a regular and pulsatile signal. While a stenotic fistula generates an altered tracing, with a reduction in amplitude, delays in the rise and fall times and asymmetry of the wave.

These variations can be used to estimate the Degree of Stenosis (DOS), which can be expressed in terms of the percentage of the narrowing of the vessel [17].

(1) $$DOS\left(\%\right)=(1-{\left(\frac{d}{D}\right)}^{2})*100$$

where DOS = Degree of Stenosis; d = stenotic vase diameter; and D = healthy vase diameter.

The devices aim to objectively evaluate the functional status of arteriovenous fistulas, vascular accesses that are essential for hemodialysis treatment but subject to frequent complications, including stenosis and aneurysms. Despite its promise, photoplethysmography for arteriovenous fistula monitoring still faces several clinical limitations that hinder its routine adoption. PPG waveforms are highly sensitive to probe–tissue contact, skin pigmentation, and local tissue thickness, resulting in significant inter- and intra-patient variability. Finally, the absence of standardized acquisition protocols and the lack of reference phantoms limit reproducibility and cross-study comparability. These challenges justify the need for a controllable, anatomically and optically realistic in vitro AVF model that enables the systematic validation of PPG-based devices under known boundary conditions. The main advantages of in vitro validation are a controllable and repeatable environment, reduced time and costs, no ethical involvement, and the possibility of recreating more scenarios without having to go through a clinical selection phase that would require more time.

In vitro testing allows us to anticipate functional issues, refine the design, reduce clinical risk and meet regulatory requirements. Its integration with in vivo validation represents a comprehensive and effective strategy that ensures safe, reliable, and clinically effective devices.

Previous studies have also explored in vitro experimental setups for PPG validation, providing useful benchmarks for the present work.

As reported by Mejía-Mejía [18], an in vitro experimental setup was built with a pulsatile pump, mockup vessels, and a fluid optically similar to blood to test PPG-based devices in controlled conditions. The validation of the setup showed the ability of the system to generate realistic PPG signals, with the possibility of varying parameters such as the heart rate and flow rate, allowing the analysis of the sensor response in different hemodynamic conditions. The artificial blood exhibited a peak absorbance at 610 nm, and the experiment was conducted by varying the stroke rate between 60 and 180 bpm (in 30 bpm increments) and the target flow between 1 and 5 L/min (in 0.5 L/min increments) Similarly, a study published in Sensors described the design and validation of pulsating phantoms made of PDMS and India ink, with artificial channels used to simulate blood vessels [19]. The setup allowed the evaluation of the PPG signal in different flow conditions, showing a high similarity with the real signals, with signal-to-noise ratios of 38.16 dB (red and infrared).

In the in vitro system developed by Nomoni et al., pressure measurements demonstrated physiologically realistic values, with peak pressures of approximately 122 mmHg upstream of the phantom and 97 mmHg downstream when the heart rate was set to 60 bpm and the flow rate to 7 L/min, confirming that mean arterial pressure remained stable across different heart rates but was predominantly influenced by flow rate changes.

Other optical tissue phantoms are reported in the literature with the aim of creating effective validation tools or to study the interaction of light with human tissues [20,21,22,23,24,25].

The pulsatile phantom developed for studying light–tissue interaction generated a pressure wave that closely approximated the human physiological range (131/78 mmHg) [20].

Chen et al. reported that for both types of gels, the Young’s modulus depended on both gel concentration and strain rate, yielding values that ranged from 50 to 1000 kPa [21].

The pulsatile phantom was designed to replicate physiological conditions, with a pressure change of 150 mmHg generating a 2% luminal volume change. Its material, silastic tubing, was selected for its elasticity, with a Young’s modulus of 2.1 MPa [22].

Njoum and Kyriacou [23] measured the viscosity of simulated blood, reporting values that ranged from 1.39 to 3.73 mPa·s.

These validations are crucial because they allow to refine the design of the device before moving to clinical trials, while ensuring reproducibility, robustness and accuracy in the measurement of the physiological signals of interest.

The aim of this paper is to develop an experimental setup for the in vitro verification of photoplethysmographic devices designed for the non-invasive diagnosis of the degree of stenosis in vascular accesses for hemodialysis. These devices combine PPG sensors with machine learning algorithms for improved diagnostic performance. Compared to the phantoms reported in the literature, which primarily aim to replicate human tissues with high fidelity in terms of their optical and mechanical properties, the present work introduces a novel approach by also focusing on the anatomical specificity of vascular geometries. While conventional phantoms often incorporate generic vessel shapes without reference to clinically relevant morphologies, this study uniquely mimics the complex geometry of an arteriovenous fistula. This targeted anatomical modeling represents a distinctive contribution, offering a more realistic and application-oriented platform for the testing and validation of PPG-based devices in vascular access scenarios.

## 2. Materials and Methods

### 2.1. Requirements

In defining the design requirements of the experimental setup, a key element was the need to reproduce the human physiology of vascular accesses used in patients undergoing hemodialysis as closely as possible. In particular, we focused on the realistic representation of the anatomical, hemodynamic, and pathological characteristics of the most common vascular accesses. To this end, the AV fistula was selected as the reference model, as it is one of the most commonly used types of vascular accesses in clinical practice, especially due to its high maturation rate, long functional duration, and relatively simple positioning. The model also includes the reproduction of one of the main degenerative phenomena that affect these accesses, namely, stenosis in the juxtanastomotic tract [26]. This tract, located near the anastomosis between the artery and the vein, is known to be particularly susceptible to the processes of intimal hyperplasia and pathological remodeling, which are among the main causes of fistula failure and the interruption of dialysis therapy.

In addition to the reproduction of the AVF, another priority aspect was to realistically represent the patient’s cardiovascular system, in particular with regard to the generation of a pulsatile flow physiologically comparable with that observable in vivo. For this reason, the setup was designed to include a pumping system capable of generating flow rates and pulsative frequencies comparable to the typical parameters of the human arterial system, such as systolic ejection and heart rate, with the possibility of introducing controlled variations to analyze the device’s response in different physiopathological conditions. This fistula has a structure composed of several distinct segments as follows: the pre-anastomotic arterial site, the anastomosis itself, the post-anastomotic venous tract, and the downstream segments. The point of greatest clinical interest is the juxtanastomotic tract. From a geometric point of view, the morphology of the fistula can vary depending on the anastomosis angle as follows: computational simulation studies have shown that angles greater than 46.5° are related to greater flow disturbances and therefore to an increased risk of stenosis at the injured site. Furthermore, the vascular wall at the fistula site can be altered over time as follows: the thickness of the overlying skin can vary from less than 1 mm to more than 4 mm, depending on the position and maturation of the access, and in many cases the skin becomes thin and fragile due to repeated needle accesses and venous dilation. The in vitro setup requirements can be divided into the following:

1. Optical Requirements
  These requirements shall reflect the behavior of light in real biological tissues, in particular, in skin and vascular districts, in order to accurately simulate the absorption, scattering, and reflection conditions that characterize the interaction between light and blood or the surrounding tissues. The main reference optical parameters, extracted from the scientific literature [27,28], useful for the design and calibration of the phantom and for the interpretation of the PPG signal, are reported below (see Table 1 and Table 2).
2. Mechanical Requirements
  Mechanical properties directly influence the response of the vascular wall to pulsed hemodynamic stimuli, as well as the ability of the tissue to withstand repeated pressure stresses, as occur during dialysis cycles. The following table (see Table 3) summarizes the main mechanical properties of the arteriovenous fistula tissue.
3. Geometric Requirements
  According to the National Kidney Foundation guidelines, the diameter of the radial artery proximal to the anastomosis site is on average 4 mm, while the diameter of the dilated cephalic vein (healthy fistula) must be at least 6 mm for the fistula to be considered mature and functional for vascular access. A particularly critical point in the morphology of the fistula is represented by the juxtanastomotic tract located approximately 2–3 cm from the anastomotic site.
4. Hydraulic and Hemodynamic Requirements
  In arteriovenous fistulas, systolic and diastolic blood pressure values play a decisive role in defining the hemodynamic conditions to which the vascular access is subjected.
  These two pressure components determine the pulsatile pressure in the fistula and affect the mechanical stress exerted on the venous wall, influencing its structural remodeling. In particular, high systolic pressure can contribute to the development of post-anastomotic aneurysms or intimal hyperplasia, while excessively low diastolic pressure can reduce the effectiveness of arterial filling and alter distal perfusion.
  C.-Y. Yang [26] provides a detailed analysis of the pressure distributions in the arteriovenous fistula, specifying the pressure value in each tract through FEA simulation (see Table 4).
5. Rheological Requirements
  Blood has a higher density than water and its viscosity depends on the flow [29]. The main properties are summarized in the following table (Table 5).

### 2.2. Benchtop Concept

**Figure 1:**
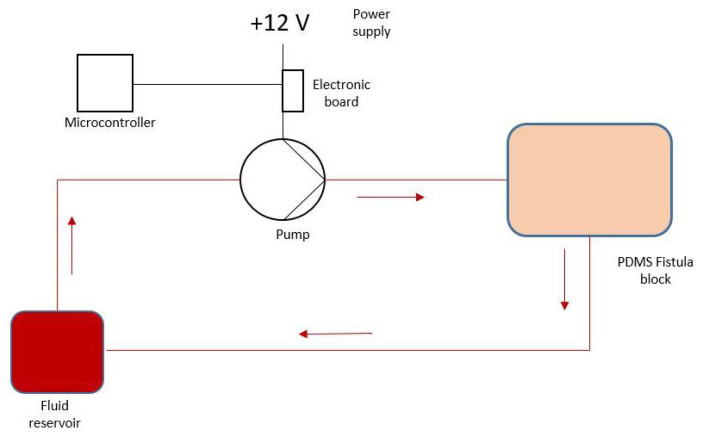
Benchtop scheme.

The verification setup is composed by a silicone block containing the channels representing the fistulas, a fluid pumping system, and a pump control system, composed of an Arduino board (Arduino Srl, Italy) and an additional electronic circuit to supply the control current necessary for the pump. The scheme of the benchtop is presented here (see Figure 1).

The pump generates a pulsatile flow of fluid that flows through the block containing the artificial fistulas, which have occlusions of 0%, 25%, 50%, and 75%, respectively. The increasing degree of stenosis allows us to simulate a progressive pathological degeneration of the vascular access. Each fistula can be individually connected to the circuit for the PPG signal acquisition phases.

### 2.3. Fistula Anatomy

**Figure 2:**
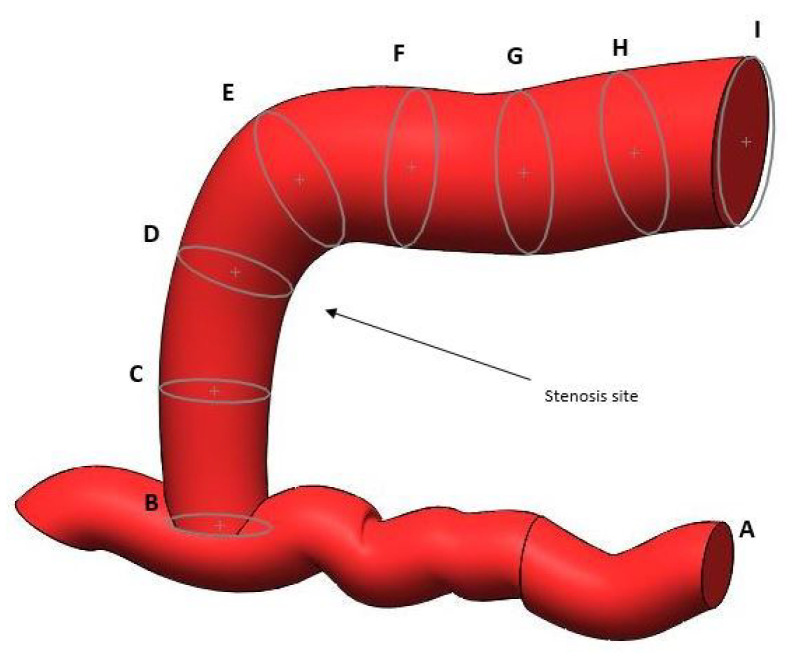
AVF cross-sectional areas.

**Figure 3:**
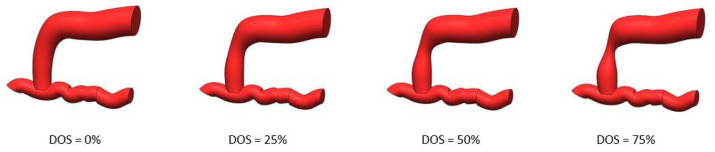
AVF degeneration.

The anatomy of the fistula faithfully reproduces the ultrasound images reported by H. Northrup [30]. The analysis presented in the paper contains all the geometric information necessary (cross-sectional lumen area with respect to the distance from the anastomotic site) to reproduce a healthy fistula and its subsequent degenerative stages (see Table 6). Three-dimensional modeling software Solidworks^TM^ (Dassault Systèmes, USA, Massachusetts) was used to develop the three-dimensional design presented here (see Figure 2 and Figure 3).

### 2.4. Material Selection

As reported by R.Saager [24], PDMS is ideal for mimicking human tissue since it has comparable optical properties. This material is generally used in optical phantoms for testing photoplethysmographic devices. To better mimic the scattering properties of biological tissues, it is also recommended to add aluminum oxide powders at various grit sizes [31].

The fistulas were designed using 3D modeling CAD software, respecting the geometries and dimensions reported in the literature. Subsequently, the fistulas were 3D printed and used to create the cavities for the PDMS molds (Reschimica Srl, Florence, Italy).

The artificial blood has been mimicked with India ink (Winsor & Newton, London, England) since this material offers an optical profile that is particularly suitable for photoplethysmographic applications, resulting in one of the best candidates for imitating the absorption of the corpuscular component of blood, particularly in the wavelengths typical of the PPG signal (red and infrared) [32].

An immersion pump was chosen, a model capable of guaranteeing a flow comparable to that of fistulas (600 mL/min). To ensure a pulsating flow, a microcontroller and a PWM signal were used. Thanks to a PWM signal, the microcontroller is able to vary the flow and the frequency, simulating physiological blood flow (600 mL/min) and heart rate (70 bpm). The artificial fistula in PDMS was connected to the volumetric pump via silicone tubes. This study exclusively used in vitro experimental models; therefore, institutional ethical approval and informed consent were not required.

## 3. Results

### 3.1. Requirements Verification

The requirements outlined in the previous section were verified using calibrated measurement instruments.

Vessel dimensions, measured with a calibrated caliper, confirmed adherence to the design specifications within a tolerance of ±0.1 mm.

The hardness measurements were performed using a durometer (Mitutoyo Europe GmbH, Neuss, Germany) that was applied to different regions of the phantom’s surface, and the average Shore A hardness value was recorded, confirming the physiological value of 20.3 ± 1.1 (95% CI: 19.8–20.8) sHA.

Tensile testing was conducted to evaluate the Young’s modulus (stiffness) of the PDMS phantom. Samples were prepared in accordance with ASTM D412 standards, cut into dog-bone specimens. A universal testing machine (Illinois Tool Works Inc., Glenview, IL, USA) was used to perform uniaxial tension tests at a rate of 10 mm/min. The stress–strain curves were obtained, and the Young’s modulus was calculated from the linear region of the stress–strain curve, yielding a value of 10.4 ± 0.9 (95% CI: 10.0–10.8) MPa, which is consistent with soft tissue mimetics. Similarly, the compression modulus was found to be 105 ± 7 (95% CI: 101.9–108.1) MPa.

The stress and strain at break were determined from the tensile tests by recording the maximum stress and strain values just before failure of the phantom material. The measured value for both parameters were within 3% of error.

The burst pressure, which refers to the maximum internal pressure the phantom can withstand before failure, was determined by pressurizing the phantom, and a pressure transducer was used to monitor the internal pressure until rupture occurred, indicating the burst pressure threshold. In fact, in accordance with the ISO 7198:2016—Cardiovascular Implants–Tubular Vascular Prostheses Regulation, a pressure value higher than 2000 mmHg was confirmed (2150 ± 120 mmHg 95% CI: 2097–2203).

Compliance was measured by applying a known pressure to the phantom and recording the corresponding volume expansion. The compliance (C) was calculated using the following formula:

(2) $$C=\frac{\Delta V}{\Delta P}$$

where ΔV is the volume variation, and ΔP is the pressure variation. The result was 2.4 ± 0.2, in line with the expected range for this type of application.

The flow verification was performed using a stopwatch and a precision scale. For flow settings of 400, 600, and 700 mL/min, the measured flow showed <1% deviation from the target values (400 ± 0.6 mL/min, 600 ± 1.2 mL/min, and 700 ± 2.3 mL/min).

Systolic and diastolic pressures were measured using a pressure transducer inserted into the pumping circuit. The pressures were recorded during the pulsatile flow cycle, with systolic pressure occurring at the peak of the pulse and diastolic pressure at the lowest point. The pressure transducer was calibrated before use to ensure accurate readings. Both measured values were found within the physiological range indicated in the hemodynamic requirements in the previous section.

The viscosity measurement was performed using a Ford cup; the results obtained confirm the correct rheological properties of the simulated blood (3.9 ± 0.3 mPa·s).

### 3.2. Signal Acquisition

Signal acquisitions were performed using a MAX 30102 optical sensor and a ESP32 (Espressif Systems, Shanghai, China). The sensor was applied directly on the juxtanastomotic tract, inside which the simulated blood flowed.

The acquisitions were performed on all four fistulas, each representing a different degree of stenosis.

To acquire data, a strategy was adopted to build a broad and diverse dataset that realistically represents the physiological and environmental variables that could affect the PPG signal in vivo conditions. Controlled variations were introduced during experimental sessions to simulate different clinical scenarios. The implemented variations included the following:

Different Heart Rates: 72, 84, 100, and 112 bpm, covering normal to tachycardic heart rates.

Ambient Light Variations: Natural light, partial darkness, and direct artificial light

Muscle Tremor Simulation: A vibrating device was placed near the PDMS model and sensor to simulate vibrations from patient tremors and assess signal sensitivity.

Secondary Stenosis Simulation: Mild obstructions were introduced before and after the main model by externally compressing the tubing to test the sensor’s response to non-localized flow disturbances.

Limb Inclination Effect: The PDMS structure was tilted by about 45 degrees while keeping the sensor in contact to simulate signal variability due to arm position.

Variation in Applied Pressure: A controlled weight was placed on the sensor to mimic changes in contact pressure during manual application and evaluate its effect on signal quality.

To enhance internal dataset variability, ten different configurations combining the above variables were used. For each, five 30-second recordings were acquired. In total, 50 recordings were collected for each occlusion level, resulting in 200 traces for analysis.

**Figure 4:**
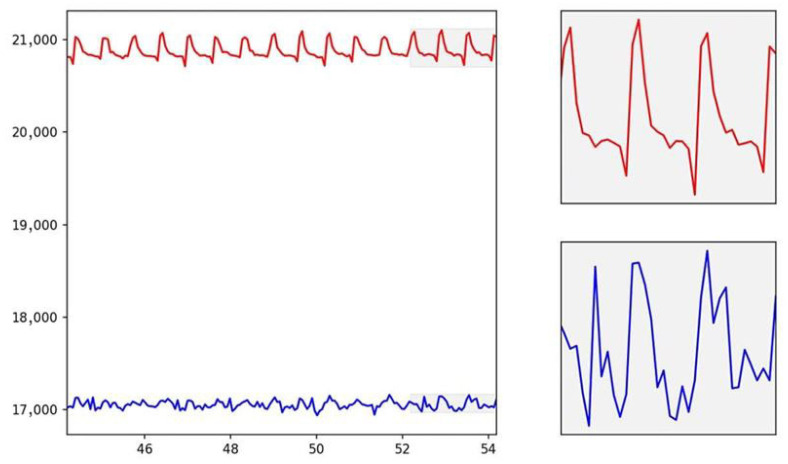
In vitro PPG signals (red line = red light, blue line = infrared light).

The figure (see Figure 4) shows the interval of the acquired trace, both in the red and infrared bands.

As can be seen, the photoplethysmographic signal reflects the physiological trace, i.e., the one acquired in vivo on real subjects. The photoplethysmographic signal obtained from the PDMS phantom was carefully compared to in vivo PPG signals to ensure its fidelity in replicating the physiological dynamics of blood flow. The phantom was designed to mimic the optical properties of human tissue, allowing the light interaction within the PDMS structure to simulate the absorption and scattering processes that occur in biological tissues. The red ink used as a blood surrogate in the phantom closely matches the optical characteristics of human blood, particularly in the red and infrared wavelengths typically used in PPG measurements.

The PPG signal obtained from the phantom (see Figure 4) exhibited the same characteristic components found in in vivo signals, including the AC (pulsatile) and DC (baseline) components. The AC component, representing the pulsatile changes in blood volume with each cardiac cycle, was clearly visible in the signal due to the well-defined flow characteristics of the blood-simulating fluid under varying pressure conditions. The DC component, associated with tissue perfusion and non-pulsatile blood volume, remained stable throughout the measurements, reflecting the relatively constant nature of tissue blood volume during the PPG recording. The mean amplitude difference between the in vitro and in vivo waveforms was 5.9 ± 3.2%, confirming a strong similarity between the two signals. In particular, the systolic upstroke and the dicrotic notch—two important features in the PPG waveform that are influenced by the arterial pressure wave and vascular elasticity—were successfully replicated in the phantom signal. These features were attributed to the mechanical properties of the PDMS phantom, which exhibited sufficient compliance and elasticity to mimic the response of human arteries to blood flow. Furthermore, variations in the flow rate and pressure within the phantom were adjusted to replicate different physiological conditions, such as those encountered in arteriovenous fistulas or during degenerative changes in the vasculature.

**Figure 5:**
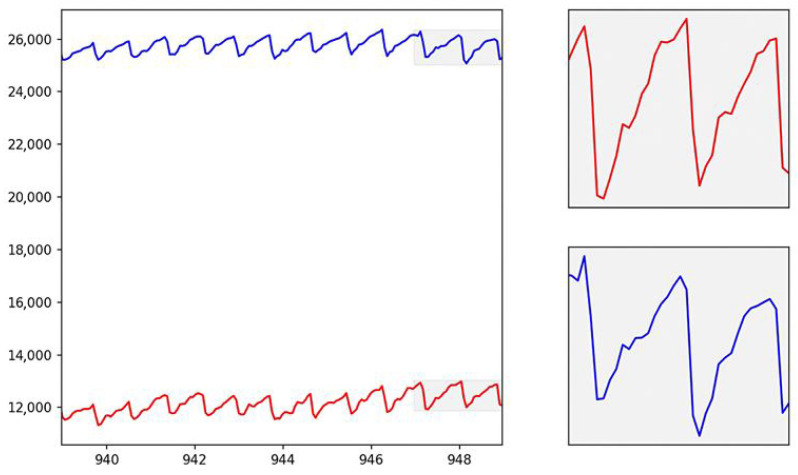
In vivo PPG signals (red line = red light, blue line = infrared light).

By tuning the pumping circuit to replicate the heart rate, stroke volume, and other relevant cardiovascular parameters, the phantom produced PPG signals with a high degree of similarity to in vivo signals recorded on a vein (see Figure 5).

## 4. Discussion

This study presents a novel experimental setup for the validation of photoplethysmographic (PPG) devices. The developed system is, to our knowledge, the first of its kind, specifically designed for the hemodialysis field, directly addressing a critical gap in the preclinical testing of vascular access monitoring devices. Current simulators used for optical sensor validation are often generic off-the-shelf tubes that lack anatomical and geometric fidelity and are primarily oriented toward oximetry validation. They fail to replicate the complex, dynamic environment of a real arteriovenous fistula (AVF), which is essential for developing reliable diagnostic tools for stenosis. In contrast, our work introduces a unique approach by focusing on anatomical and geometric specificity. The phantom was fabricated to precisely mimic the complex geometry of an AVF, including various degrees of stenosis. This capability to reproduce specific pathological morphologies and controlled flow conditions represents a significant advance. The quantitative validation confirms that the phantom’s properties are highly realistic and suitable for device testing. The anatomical dimensions were fabricated with a tolerance of 0.1 mm. In particular, the measured Shore A hardness of 20.3 ± 1.1 sHA is consistent with values reported in the literature (Kriener et al. [33] reported hardness values in the range 13.3–28.0 sHA). The measured Young’s modulus of 10.4 ± 0.9 MPa is comparable to those reported in the mechanical characterization studies of the human body across different anatomical regions, which ranged between 4.02 and 83.3 MPa [34]. The compression modulus (105 ± 7 MPa) also aligns with those reported by van Kuilenburg [35] (100 MPa), confirming the physiological realism of the material.

Its burst pressure of 2150 mmHg exceeds the required ISO standards, demonstrating robustness. From a hemodynamic standpoint, the system accurately reproduces physiological flow. The flow rates between 400 and 700 mL/min showed an error of less than 1% with respect to the typical flow rates observed during hemodialysis [36]. Meanwhile, the simulated blood viscosity of 3.9 mPa·s is in line with clinical values [29]. The system successfully generated physiological pressures and realistic PPG waveforms, confirming its ability to serve as a reliable platform for evaluating sensor performance under known, controlled conditions. These results underscore the physiological relevance of our setup, validating its potential to accelerate the development of clinically effective PPG-based monitoring devices.

As with any experimental platform, the proposed setup has certain limitations that should be acknowledged. There is limited availability in the literature of three-dimensional reconstructions of pathological arteriovenous fistulas (e.g., stenotic or aneurysmal geometries), which constrains the anatomical diversity that can currently be reproduced with our approach. Moreover, although the phantom’s optical and mechanical properties approximate those of vascular tissue, the scattering behavior could be reproduced more faithfully by adding particulate scatterers to the blood surrogate. Finally, the current model is a single-layer structure; future developments will focus on multilayer fabrication techniques to better mimic the layered composition of the vessel wall and improve both anatomical and functional fidelity.

Future work will also aim to enhance the optical and structural realism of the phantom. A key improvement will be the incorporation of titanium oxide and aluminum oxide powders with carefully selected particle sizes and controlled spatial distribution, which are expected to improve the phantom’s light-scattering properties and enable the more accurate testing of PPG devices. Additionally, extending the present setup to different types of vascular accesses—such as brachiocephalic or basilic fistulas, as well as pathological variants including bifurcations, accentuated curvatures, or different anastomotic angles—will increase the versatility of the platform for comparative analyses. The fabrication of vascular structures using dip-coating techniques will further enhance the physiological fidelity of the phantom, providing a more comprehensive and reproducible platform for device evaluation.

Beyond technical development, this system offers several promising applications. It could facilitate the fabrication of realistic physical models from a comprehensive 3D image database of pathological fistulas derived from clinical CT scans, providing a highly specific and standardized platform for device testing. The platform can also serve educational purposes as follows: by reproducing realistic AVF hemodynamics, it allows medical students and clinicians to practice the signal acquisition, interpretation, and recognition of pathological changes in a safe and controlled environment. Moreover, the approach can be extended to study aneurysms and vascular degenerations in other regions of the human body and to generate large numbers of low-cost replicas to train AI algorithms under various degenerative conditions.

## 5. Conclusions

This study presents a novel in vitro experimental setup for the validation of photoplethysmographic devices targeting arteriovenous fistula monitoring. The system accurately replicates anatomical, mechanical, optical, and hemodynamic characteristics of vascular accesses, enabling controlled and reproducible PPG signal acquisition. Quantitative validation confirmed the physiological realism of the phantom, supporting its suitability for device testing. While certain limitations remain—such as restricted anatomical diversity and single-layer vessel structures—the platform provides a versatile foundation for future enhancements, including multilayer fabrication, expanded vascular geometries, and improved optical fidelity. Overall, this setup represents a robust tool for preclinical evaluation, educational purposes, and the development of clinically effective PPG-based monitoring devices.

## Figures and Tables

**Table 1:** Absorption coefficient (${\mathrm{mm}}^{-1}$).

Layer	600–700 nm	750–1000 nm
Epidermis	2–4	0.5–2.5
Dermis	2–5	2–5
Blood	0.02–0.05	0.03–0.06

**Table 2:** Scattering coefficient (mm^−1^).

Layer	600–850 nm
Epidermis	1–10
Dermis	3.2–1.2
Blood	2.5

**Table 3:** Mechanical properties.

Mechanical Parameter	Value
Hardness (ShA)	20
Young’s modulus (MPa)	9–12
Compression modulus (MPa)	100
Compliance (%)	1.6–5.9
Stress at break (MPa)	1–4.3
Strain at break (%)	59–242
Burst pressure (mmHg)	1600–3900

**Table 4:** Hydraulic and hemodynamic parameters.

Parameter	Value
Blood Flow (mL/min)	600–1500
Heart rate (bpm)	60–80
Stroke volume (mL)	70–90
Systolic pressure (mmHg)	110–130
Diastolic pressure (mmHg)	70–85

**Table 5:** Rheological parameters.

Parameter	Value at 37 °C
Density (kg/m^3^)	1050–1060
Viscosity (mPa·s)	3.5–4.5

**Table 6:** Geometric parameters.

Section Label	Distance (mm)	Cross-Sectional Area (mm^2^)	Radius (mm)
A	NA	12.57	2
B	0	15	2.2
C	5	17	2.32
D	10	20	2.52
E	15	32	3.19
F	20	35	3.34
G	25	37	3.43
H	30	38	3.48
I	35	40	3.57

## Data Availability

All data reported in the text are referenced punctually in the bibliography. The tools and materials included in this research are open source (Arduino, MAX30102, etc.) and available online or in any store.

## Abbreviations

The following abbreviations are used in this manuscript:

PPG	photoplethysmography
AVF	arteriovenous fistula
